# Energy density-driven structural tuning of Al_2_O_3_/AgO films for enhanced toxic gas detection

**DOI:** 10.1039/d5ra08204h

**Published:** 2026-05-08

**Authors:** Doaa Yaseen Doohee, Abas Azarian, Mohammad Reza Mozaffari

**Affiliations:** a Department of Physics, University of Qom Qom Iran Azarian@qom.ac.ir

## Abstract

This study examines how laser energy density during pulsed laser deposition (PLD) affects the structural, optical, electrical, and gas-sensing properties of Al_2_O_3_/AgO thin films. The films were deposited at energy densities of 10.2, 21.2, 31, and 40.8 J cm^−2^ and analyzed using XRD, FE-SEM, AFM, UV-vis, and Hall effect techniques. The results showed that increasing the energy density improves the crystallinity and conductivity up to 31 J cm^−2^, while excessive energy at 40.8 J cm^−2^ induces defects and re-evaporation, enhancing the gas sensitivity due to the increased number of active sites. The optical band gaps ranged from 1.821 to 1.967 eV, varying with the grain size. All films exhibited n-type behavior. Gas sensing tests for NO_2_ and H_2_S in the range of 40–250 °C revealed the highest sensitivity at 250 °C. The film deposited at 40.8 J cm^−2^ showed the best sensing performance due to the oxygen vacancies and nanoclusters. This study confirms that optimizing laser energy enables the tailoring of Al_2_O_3_/AgO films for enhanced gas sensor applications.

## Abstract 2

This study examines how laser energy density during pulsed laser deposition (PLD) affects the structural, optical, electrical, and gas-sensing properties of Al_2_O_3_/AgO thin films.

## Graphical abstract

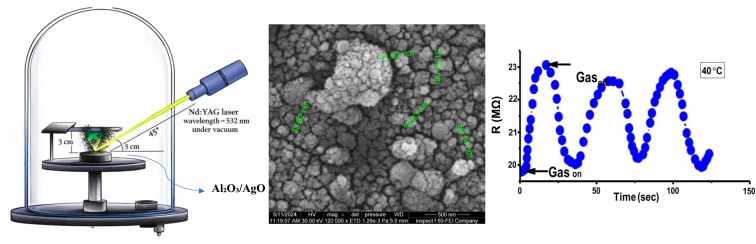

## 1 Introduction

Gas detection technologies have undergone significant and continuous advancements over the past several decades, largely driven by rapid industrialization and the increasing demand for environmental and safety monitoring. A foundational milestone in this field dates back to 1953, when Brattain and Bardeen demonstrated that gas adsorption on semiconductor materials could induce measurable changes in electrical conductance. Subsequently, the first commercial gas sensor, developed by Taguchi in 1968, was introduced for hydrocarbon detection.^1–3^ Since then, gas sensors have been extensively utilized across a wide range of applications, including environmental pollution monitoring, domestic and industrial safety, automotive emission control, air quality assessment, and, more recently, medical diagnostics, such as breath analysis.^4,5^

Various fabrication approaches have been explored to enhance sensor performance, including electrochemical and optical methods, in addition to solid-state gas sensors based on diverse sensing materials, particularly metal oxides (MOXs).^6^ In these systems, the primary sensing mechanism is governed by variations in the electrical resistance arising from the surface interactions between the sensing material and the target gas molecules. This behavior is strongly influenced by the intrinsic material characteristics, such as the composition, crystallite size, and microstructural features. Over the past six decades, substantial research efforts have been directed toward elucidating gas sensing mechanisms in both p-type and n-type semiconductors; advanced strategies, such as thermal modulation (static and dynamic), doping techniques, and micro- and nano-structuring, have been employed to optimize sensor performance.^7,8^

Among thin-film fabrication techniques, pulsed laser deposition (PLD) has emerged as a highly versatile and powerful method for tailoring the structural and functional properties of metal oxide thin films. Unlike many conventional deposition techniques, PLD provides precise control over the laser energy density, which directly determines the kinetic energy of the ablated species and consequently affects the film crystallinity, defect density, and oxygen vacancy concentration. Such control is particularly critical in gas sensing applications, where surface defects and microstructural features play a decisive role in the adsorption–reaction–desorption processes. Furthermore, PLD enables the near-stoichiometric transfer of complex oxide targets and facilitates the formation of high-purity films with well-defined microstructures. These advantages make PLD an ideal platform for investigating the structure–property relationships of sensing materials. Accordingly, PLD was employed in this study to systematically examine the influence of laser energy density on the microstructural evolution and gas sensing performance of Al_2_O_3_/AgO thin films.^9–11^

The vaporization of condensed matter through photon–matter interactions represents one of the most effective deposition approaches in solid-state physics and analytical chemistry. Pulsed laser deposition (PLD) is a prominent technique in this category, wherein a laser beam—characterized by its pulse duration, wavelength, and fluence—interacts with a bulk target material.^12^ When a high-energy, short-pulse laser irradiates the target surface, it induces rapid localized heating, leading to the transformation of the solid material into a highly energetic vapor plume composed of ions, atoms, and neutral species. This ablation plume propagates at velocities of several kilometers per second and can subsequently be deposited onto a substrate to form thin films.^13,14^

From an application perspective, the detection of hazardous gases, such as nitrogen dioxide (NO_2_) and hydrogen sulfide (H_2_S), is of paramount importance. NO_2_ is a toxic and highly reactive gas that is primarily generated from industrial processes and vehicular emissions. It plays a significant role in environmental degradation phenomena, including acid rain formation and photochemical smog, and can severely impact the human nervous system even at low concentrations, potentially leading to loss of consciousness.^15^ Similarly, H_2_S is a colorless, flammable, and highly toxic gas characterized by its distinctive odor of rotten eggs. Exposure to low concentrations may cause headaches, dizziness, and irritation of the eyes and respiratory system, while high concentrations can result in acute toxicity and fatal outcomes.^16^ Consequently, the development of reliable and efficient sensors for NO_2_ and H_2_S detection remains a critical research priority.

A key factor influencing the thin-film quality in PLD is the laser energy density used during the deposition. The input energy must be carefully optimized to ensure efficient material transfer while avoiding excessive or insufficient ablation. The energy delivered during deposition significantly affects the film growth dynamics and, consequently, the structural integrity and functional performance of the resulting thin films. One critical phenomenon associated with excessive energy input is re-evaporation, which leads to material loss from the film surface through the detachment of adsorbed atoms. This process occurs when the kinetic or thermal energy supplied to the adatoms exceeds the energy required for surface stabilization, causing them to desorb before complete thermalization. Re-evaporation is particularly pronounced under conditions of high substrate temperatures or when the substrate temperature approaches or slightly exceeds room temperature during the early growth stages, where atoms are weakly bound by van der Waals forces.^17^

The selection of Al_2_O_3_/AgO as the sensing material in this study is motivated by its synergistic structural and catalytic characteristics, which render it highly suitable for gas sensing applications. AgO acts as the active phase, providing abundant redox sites that enhance interactions with both oxidizing (NO_2_) and reducing (H_2_S) gases. In particular, the strong affinity of AgO toward H_2_S leads to surface sulfidation (Ag → Ag_2_S), resulting in a pronounced and rapid change in electrical resistance. Meanwhile, Al_2_O_3_ serves as a thermally stable and chemically inert matrix that promotes the uniform dispersion of active particles and stabilizes grain-boundary potential barriers. This combination effectively enhances sensor stability, minimizes the baseline drift, and improves the resistance to humidity-induced interference. Furthermore, the high surface-to-volume ratio associated with Al_2_O_3_ contributes to an improved adsorption efficiency, thereby enhancing the overall sensing performance.^18^

In this study, a specific gap in the existing literature is addressed by systematically investigating the influence of laser energy density in PLD on the structural, optical, and electrical properties of Al_2_O_3_/AgO thin films and correlating these properties with their gas sensing performance. The results demonstrate that increasing the laser energy density enhances the film porosity and surface roughness, thereby increasing the density of active adsorption sites and improving the sensitivity toward NO_2_ and H_2_S gases. Although previous studies have predominantly focused on single oxides or simple composite systems without thoroughly examining the role of deposition energy, the present study highlights that precise control of the PLD parameters enables effective tuning of the grain size, crystallinity, oxygen vacancy concentration, and porosity. This approach provides a practical and scalable pathway for designing high-performance gas sensors that combine enhanced sensitivity and excellent thermal stability.

## 2 Experimental methods

### 2.1 Preparation of the Al_2_O_3_/AgO solid target

A mixture was prepared by combining 0.2 g of silver oxide (AgO, 99.5% purity, Merck Company, German) with 0.8 g of aluminum oxide (Al_2_O_3_, 99% purity, Fluka Company, Swiss), yielding a weight ratio of 1 : 4 (AgO/Al_2_O_3_). The powder mixture was thoroughly homogenized and subsequently heated in a controlled-temperature oven at 120 °C for 2 hours to enhance material interaction. After the thermal treatment, the mixture was allowed to cool naturally to room temperature. It was then subjected to uniaxial pressing under a pressure of 5 tons to form a compact solid target with a diameter of 1.5 cm and a thickness of 0.5 cm, which is suitable for use in pulsed laser deposition (PLD) processes.

### 2.2 Mask preparation

**Fig. 1:**
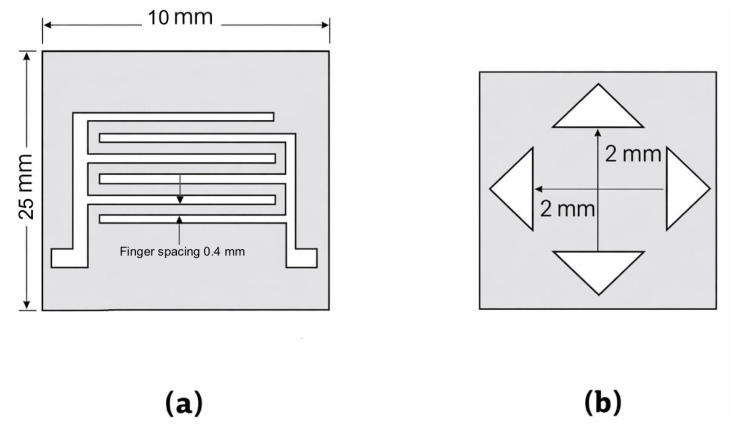
Mask of (a) gas sensor and (b) Hall effect.

As illustrated in Fig. 1, specially designed aluminum foil masks were fabricated and thoroughly cleaned using distilled water and alcohol to ensure high surface purity and an accurate pattern definition. These masks were employed to precisely define the electrode geometry required for different electrical measurements. Specifically, mask (a) was utilized for gas sensing measurements and current–voltage (*I*–*V*) characterization, while mask (b) was designed for Hall effect measurements.

To fabricate the gas sensor electrodes, the glass substrates were initially subjected to rigorous cleaning procedures to eliminate surface contaminants and ensure optimal film adhesion. A thermal evaporation system was then employed to deposit aluminum (Al) electrodes. In this process, high-purity aluminum was placed in a crucible within a vacuum chamber evacuated to a pressure of approximately 10^−5^ torr. The source material was resistively heated until evaporation occurred, generating a vapor flux that propagated through a vacuum environment and condensed onto the substrate surface, forming a uniform thin film.

The thickness of the deposited film was continuously monitored using a quartz crystal microbalance (QCM) to ensure precise thickness control. Once the desired thickness of approximately 200 nm was achieved, the heating process was terminated, and the system was allowed to cool gradually to room temperature before retrieving the coated substrates. The resulting electrode configuration, as shown in Fig. 1(a), provided well-defined conductive pathways suitable for subsequent thin-film deposition and gas sensing measurements.

In the case of Hall effect measurements, as depicted in Fig. 1(b), aluminum foil masks with well-defined geometrical dimensions were fabricated to ensure measurement accuracy and reproducibility. These masks were similarly cleaned using distilled water and alcohol to remove any contaminants that could affect the electrode pattern fidelity. The use of precisely defined masks ensured a uniform current distribution and reliable electrical characterization during the Hall effect analysis.

### 2.3 Preparation of the Al_2_O_3_/AgO thin films

**Fig. 2:**
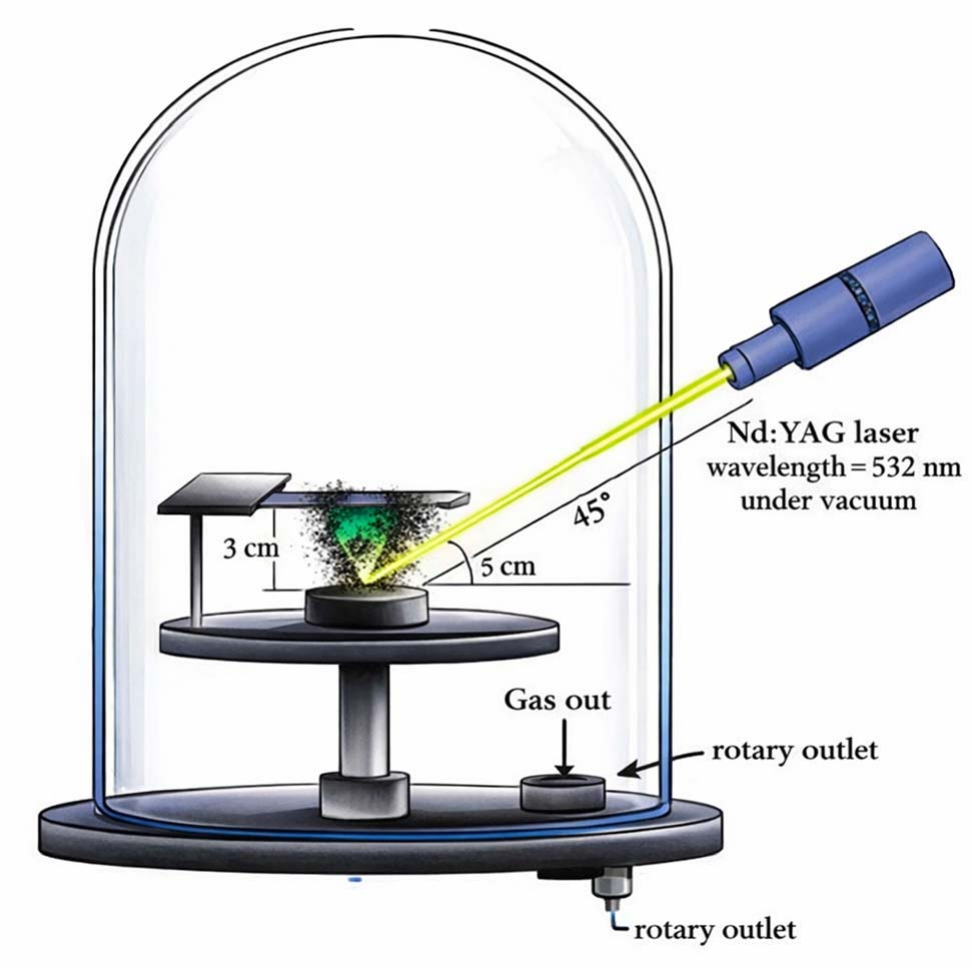
Schematic of the pulsed laser deposition (PLD) experimental setup used for thin film deposition, showing the Nd:YAG laser source, target, substrate, and geometrical configuration inside the vacuum chamber.

Thin films of Al_2_O_3_/AgO were deposited onto clean substrates of glass using the pulsed laser deposition (PLD) technique. The deposition process was carried out at room temperature inside a vacuum chamber maintained at a pressure of 0.01 mbar. A Q-switched Nd:YAG laser was employed as the energy source, operating at varying energy densities (10.2, 21.2, 31.0, and 40.8 J cm^−2^) to study their effect on film properties. The laser beam was focused and directed onto the surface of the Al_2_O_3_/AgO target at an incidence angle of 45° (Fig. 2), ensuring efficient ablation. The glass substrates were positioned directly in front of the target at a distance of 3 cm, with their surfaces aligned parallel to the target surface, to ensure uniform film deposition.

The diameter of the laser spot on the target surface was approximately 2.5 mm. The laser operated in the Q-switched mode with a pulse repetition rate of 4 Hz. During the deposition process, the target holder maintained the ablation target fixed in the vertical orientation. To produce consistent ablation, the target, which is typically disk-shaped, must be rotated throughout the deposition. The target can be moved along both the *x*- and *y*-axes, while the laser interaction spot remains stationary using a stepper motor. To achieve a longer time, the target surface area can be used more efficiently, without the formation of holes.

### 2.4 Characterization

The structural, morphological, optical, and electrical characterizations of the prepared Al_2_O_3_/AgO thin films were carried out using several complementary techniques. X-ray diffraction (XRD) measurements were performed using an AERIS diffractometer (Malvern PANalytical, Netherlands) operating with Cu Kα radiation (*λ* = 1.5406 Å) at 40 kV and 15 mA. The data were collected over a 2*θ* range of 20–80° with a step size of 0.02° and a scan speed of 2° min^−1^. Surface topography was investigated by applying atomic force microscopy (AFM, TT-2, American workshop) in tapping mode, operated in tapping mode with a standard Si cantilever having a nominal tip radius of <10 nm. The scans were typically acquired over an area of 2 × 2 µm^2^ to evaluate surface roughness and grain distribution. Morphological and microstructural features were further examined using field emission scanning electron microscopy (FESEM, Malvern PANalytical) operated at an accelerating voltage of 30 kV, which provided high-resolution images of the thin-film surface. Optical properties were analyzed using UV-visible spectrophotometry (Shimadzu UV-1900i, Japan) in the wavelength range of 190–1100 nm, allowing for the determination of absorbance, transmittance, and bandgap energy. Electrical characterization was carried out using a Hall effect measurement system (HMS-3000, Ecopia, Korea). For this purpose, silver electrodes were deposited on the films to ensure ohmic contact. For laser processing, a Q-switched Nd:YAG laser (Guangzhou Danye Optical Co., Ltd, China) with a wavelength of 532 nm and a pulse duration of ∼10 ns was employed.

### 2.5 Gas sensor

The gas sensing performance of the prepared Al_2_O_3_/AgO thin film samples was systematically evaluated using a custom-designed experimental setup. The measurement system comprised electrode-integrated sensor devices for electrical signal acquisition, along with calibrated temperature and pressure controllers to ensure precise monitoring and stability of the testing environment. Dry air was employed as the carrier gas to provide a stable baseline and minimize interference from ambient humidity. For each sensing cycle, the gas injection and purging intervals were accurately controlled and time-stamped to guarantee the repeatability and consistency of the measurements. Prior to gas exposure, the sensors were stabilized under constant air flow to establish reliable baseline resistance. The sensing characteristics were investigated at three operating temperatures (40 °C, 150 °C, and 250 °C) in order to evaluate the effect of thermal activation on adsorption–desorption kinetics and charge transfer mechanisms. The sensors were then exposed to fixed concentrations of target gases, specifically 150 ppm of nitrogen dioxide (NO_2_) and 200 ppm of hydrogen sulfide (H_2_S). These concentrations were selected to represent typical levels relevant to environmental monitoring and industrial safety applications. The dynamic response of the sensors was recorded in terms of resistance variation upon gas exposure and recovery, enabling a detailed analysis of sensitivity, response/recovery behavior, and the influence of laser energy density on gas sensing performance.

## 3 Results and discussion

**Fig. 3:**
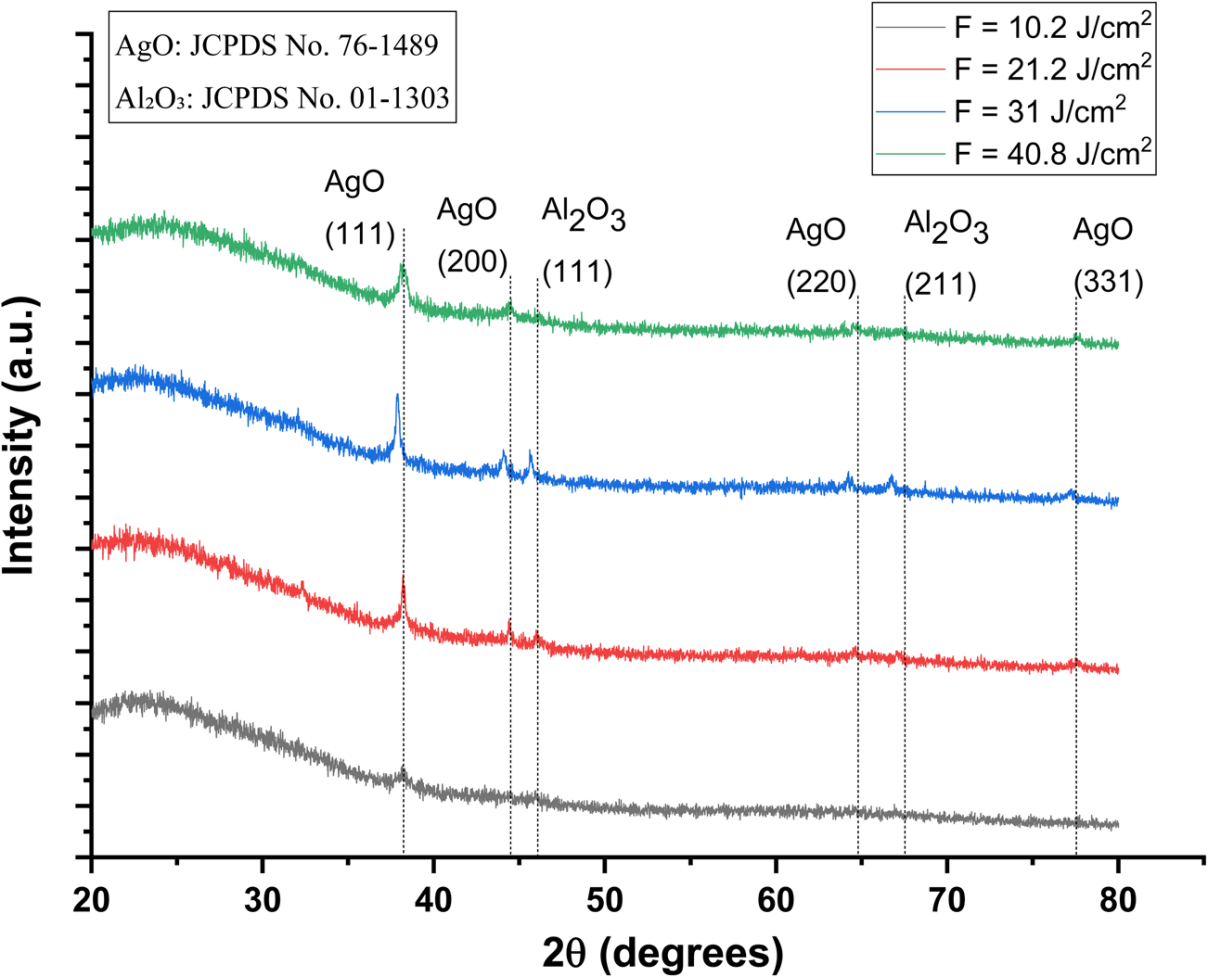
X-ray diffraction patterns of the Al_2_O_3_/AgO samples at different energy densities of 10.2, 21.2, 31, and 40.8 J cm^−2^.

Fig. 3 represents the X-ray diffraction (XRD) pattern of the Al_2_O_3_/AgO thin films deposited on glass substrates and prepared by pulsed laser deposition at different energy densities (10.2, 21.2, 31, and 40.8 J cm^−2^). The films are polycrystalline structures. For all energy densities, XRD patterns possess many diffraction peaks, which indicate the formation of polycrystalline AgO with planes (111), (200), and (220), (JCPDS No. 76-1489), and polycrystalline Al_2_O_3_ with planes (111) and (211) (JCPDS No. 01-1303), which is consistent with the study by Kourosh Motevalli and his group.^19^

At the investigated laser energy densities (10.2, 21.2, 31, and 40.8 J cm^−2^), the XRD patterns indicate the formation of Al_2_O_3_/AgO composite thin films with a relatively weak appearance of the Al_2_O_3_ phase. This behavior can be attributed to the absence of a strong interphase interaction between the Al_2_O_3_ and AgO structures at lower laser energies. In addition, the weak intensity of some AgO diffraction peaks is likely related to the partial shielding or absorption effect caused by the Al_2_O_3_ component during the laser–target interaction, which reduces the effective ablation of AgO species.^20^ As the laser energy density increases, the deposition dynamics during the PLD process change significantly. Higher laser fluence enhances the ablation rate of the target material, resulting in a larger flux of energetic species (atoms, ions, and clusters) reaching the substrate surface. These species possess higher kinetic energy, which increases their surface mobility and promotes more efficient surface diffusion during film growth. Consequently, the atoms can more readily migrate to energetically favorable lattice sites, facilitating improved atomic ordering and crystal growth while reducing structural disorders. This process also enhances the coalescence of small nuclei and adjacent grains, leading to larger crystallites and a reduction in grain boundary density. This effect becomes particularly noticeable at a laser energy density of 31 J cm^−2^, where a shift in the characteristic AgO diffraction peaks is observed. These peak shifts suggest the formation of an interphase or structural interaction between the Al_2_O_3_ and AgO phases, indicating the development of a modified composite structure rather than a simple physical mixture of the two oxides. The observed peak displacement also suggests partial incorporation between the two phases, which can modify the crystallographic arrangement of the deposited film. As a result, a new composite structure corresponding to the Al_2_O_3_/AgO system is formed, which is consistent with the structural evolution observed in the XRD patterns. However, when the laser energy density increases further to 40.8 J cm^−2^, additional high-energy processes, such as re-evaporation and defect formation, begin to occur. At this stage, the excessive kinetic energy imparted to the growing film may lead to partial re-evaporation of the material and the formation of structural defects, which can slightly reduce the effective crystallite size or modify the crystalline order.^21^

Overall, the improvement in crystallinity with increasing laser energy density can therefore be explained by the combined effects of enhanced ablation efficiency, increased kinetic energy of the arriving species, improved surface diffusion of adatoms, and coalescence of small nuclei into larger crystallites. These mechanisms collectively reduce grain boundary density and promote the formation of a more ordered crystalline structure, as reflected in the XRD parameters summarized in Table 1.

**Table 1:** XRD parameters of the Al_2_O_3_/AgO thin films

Energy density (J cm^−2^)	2*θ* (°)	FWHM (°)	*D* (nm)	*D*_ave_ (nm)
*F* = 10.2	38.173	7.26396	1.158	1.487 (poorly crystalline)
	45.939	6.61913	1.304	
	66.971	4.76297	2.000	
*F* = 21.2	38.162	2.10298	3.999	7.235
	44.366	2.662	3.224	
	45.961	3.61108	2.391	
	64.573	3.872	2.428	
	67.015	0.68683	13.876	
	77.520	0.5826	17.491	
*F* = 31	37.953	0.70212	13.300	9.703
	44.157	2.43792	3.909	
	45.730	1.42301	6.735	
	64.265	1.63219	6.388	
	66.773	0.90392	11.699	
	77.289	0.6984	16.186	
*F* = 40.8	38.085	2.15759	4.330	5.781
	44.443	3.6649	2.603	
	64.551	5.7292	1.823	
	77.509	0.78794	14.369	

**Fig. 4:**
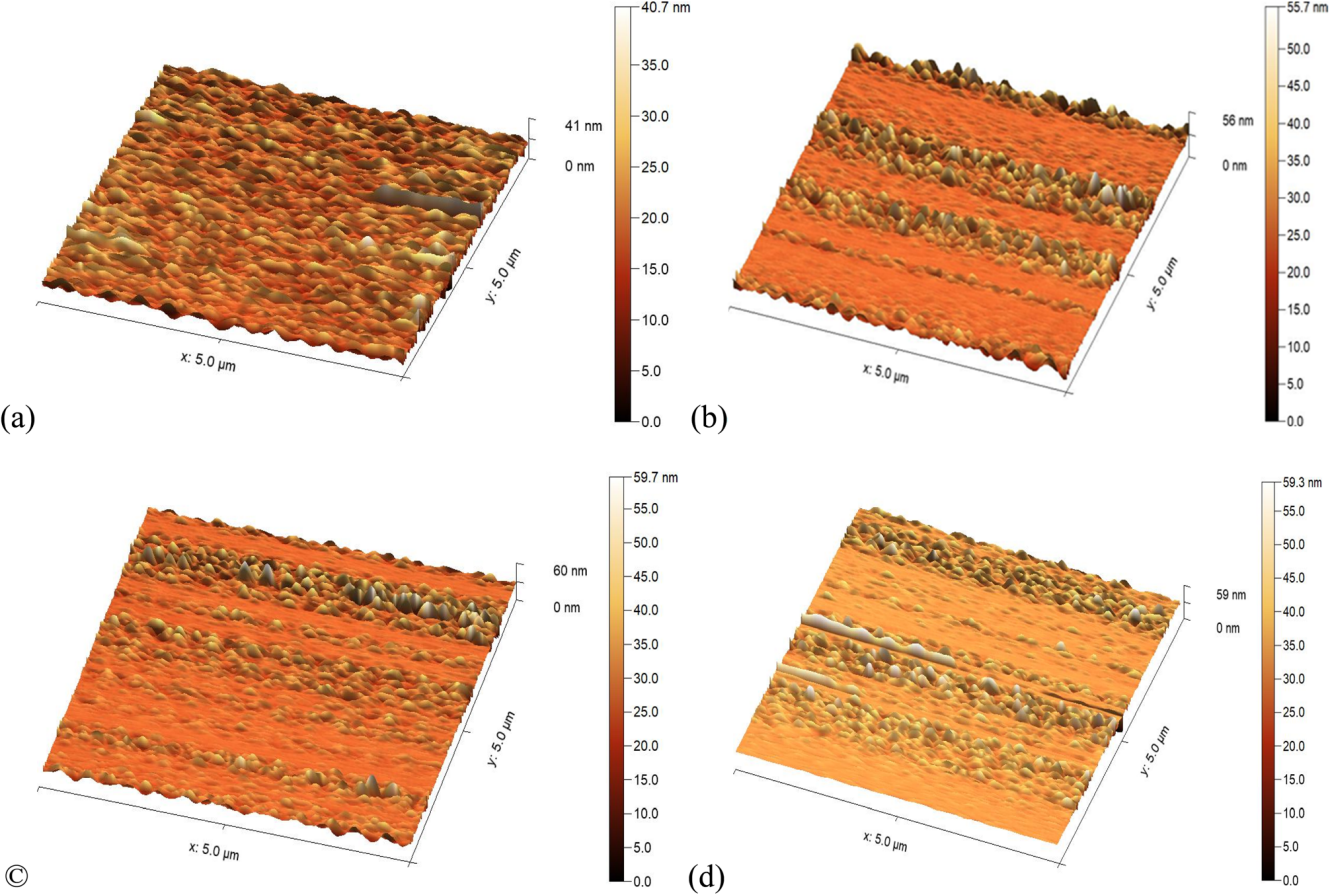
AFM results of the Al_2_O_3_/AgO samples at different energy densities of (a) *F* = 10.2 J cm^−2^, (b) *F* = 21.2 J cm^−2^, (c) *F* = 31 J cm^−2^, and (d) *F* = 40.8 J cm^−2^.

Fig. 4 shows 3D images of the surface of Al_2_O_3_/AgO thin films deposited on glass substrates prepared using the PLD method at different energies. It appears that the average roughness of the films increases with the increasing energy density of the laser. When the energy density of the laser increases, the number of atoms emitted from the target increases, and the energy of the emitted atoms increases. This leads to Al_2_O_3_/AgO films with larger grains on the surface of the substrate, which increases the surface roughness, as shown in Table 2. At a higher energy density (40.8 J cm^−2^) for Al_2_O_3_/AgO, there may be an improvement in the balance between the number of atoms or molecules that reach the substrate and settle there and the number of atoms that re-evaporate, which leads to a reduction in roughness compared to the energy density of 31 J cm^−2^.^22^

**Table 2:** Average grain size, roughness rate, and root mean square roughness of the Al_2_O_3_/AgO thin films according to AFM measurements

Materials	Energy density (J cm^−2^)	Average grain size	RMS roughness	Mean roughness
Al_2_O_3_/AgO	10.2	17.23 nm	2.93 nm	2.10 nm
	21.2	25.46 nm	3.61 nm	2.32 nm
	31	27.19 nm	3.87 nm	2.54 nm
	40.8	32.47 nm	3.95 nm	2.52 nm

**Fig. 5:**
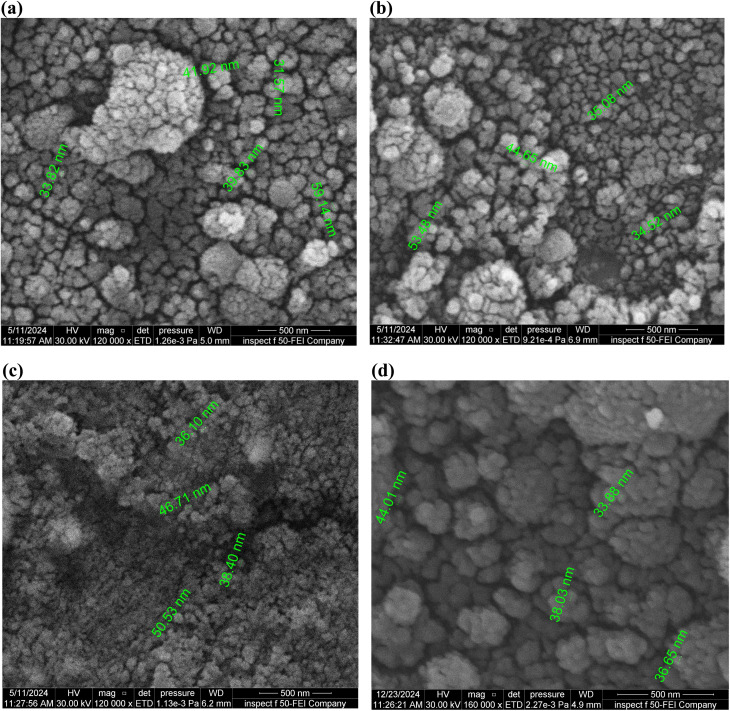
FE-SEM images of the Al_2_O_3_/AgO samples at different energy densities of (a) *F* = 10.2 J cm^−2^, (b) *F* = 21.2 J cm^−2^, (c) *F* = 31 J cm^−2^, and (d) *F* = 40.8 J cm^−2^.

Fig. 5 and Table 3 present FE-SEM top-view images and particle size analysis of Al_2_O_3_/AgO thin films deposited at laser energy densities of 10.2, 21.2, 31, and 40.8 J cm^−2^. To ensure reliable comparison, the particle size analysis was carried out using the ImageJ software by measuring multiple representative particles (typically 4–6 particles from different regions of each sample) to obtain an approximate statistical evaluation of the surface morphology. The calculated particle sizes are reported in Table 3 as average values with standard deviations (mean ± standard deviation (SD)), providing a more accurate representation of the size distribution. The relatively higher standard deviation at lower energy densities indicates non-uniform particle distribution, while the reduced deviation at higher energy densities reflects improved uniformity and surface homogeneity. With increasing laser energy, the films exhibit larger and more irregular surface features, reduced interparticle bonding, and higher porosity.^22^ Importantly, these surface features correspond to agglomerates, *i.e.*, clusters formed from multiple nanocrystalline grains, which are naturally larger than the crystallite sizes determined by XRD. Therefore, the particle sizes obtained from FE-SEM images should be considered approximate estimations of agglomerate dimensions rather than exact crystallite sizes.

**Table 3:** Average particle sizes of the Al_2_O_3_/AgO thin films deposited at different laser energy densities, calculated from FE-SEM images using the ImageJ software. The values are presented as the mean ± standard deviation based on the measurements of multiple representative particles from different regions of each sample. The reported particle sizes correspond to the agglomerates and provide an approximate estimation of surface morphology

Energy density (J cm^−2^)	Al_2_O_3_/AgO
10.2	41.26 ± 10.86 nm
21.2	41.96 ± 8.99 nm
31	42.94 ± 6.81 nm
40.8	38.80 ± 4.28 nm

At the highest fluence of 40.8 J cm^−2^, atomic rearrangement and re-evaporation effects reduce the average size of individual grains while simultaneously promoting the formation of spherical nanoclusters and visible agglomerates. These morphological changes are expected to enhance gas sensitivity due to the combined effects of increased porosity, higher surface energy, and larger effective surface area—factors widely recognized as beneficial for sensor and photodetector applications.^23^ In Al_2_O_3_/AgO thin films, distinct Al_2_O_3_ crystals are visible at energy densities of 10.2 and 21.2 J cm^−2^, while at 31 J cm^−2^, the reduced clarity suggests interphase formation between Al_2_O_3_ and AgO, which is consistent with the peak shifts observed in the XRD patterns. This energy density can therefore be considered a threshold for structural modification. At 10.2 J cm^−2^, the laser fluence is insufficient to induce interphase formation, resulting in weak oxide features. In contrast, at 40.8 J cm^−2^, although Al_2_O_3_ reflections become more pronounced, excessive re-evaporation and defect generation inhibit the formation of a stable interphase between the two oxides.

**Fig. 6:**
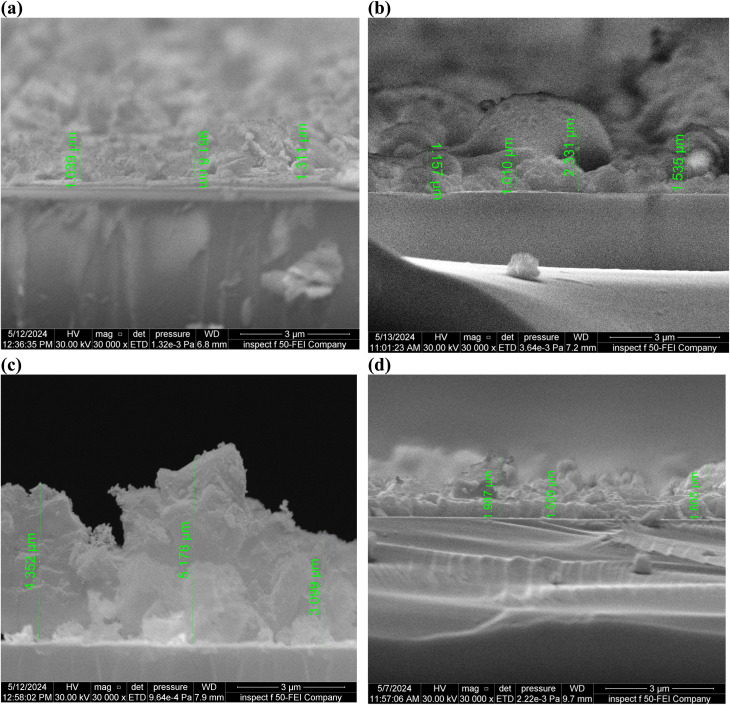
Cross-section FE-SEM images of the Al_2_O_3_/AgO films at different energy densities of (a) *F* = 10.2 J cm^−2^ (b) *F* = 21.2 J cm^−2^, (c) *F* = 31 J cm^−2^, and (d) *F* = 40.8 J cm^−2^.

Fig. 6 shows the variation in thin film thickness with increasing laser energy density for Al_2_O_3_/AgO thin films, as summarized in Table 4. The observed increase in thickness at lower to intermediate energy densities can be attributed to the enhanced energy transferred to the target atoms during the pulsed laser deposition (PLD) process. This increased energy leads to a more effective ablation process, resulting in a higher number of atoms, ions, and clusters being ejected from the target and subsequently deposited onto the substrate. Consequently, the accumulation of these species contributes to an increase in film thickness.^24^ At a higher energy density of 40.8 J cm^−2^, a reduction in film thickness is observed, as indicated in Table 4. This behavior can be explained by the onset of the ablation threshold, where the laser energy exceeds the optimal range required for efficient material transfer. Under such conditions, excessive energy input leads to intense evaporation and scattering of the ablated species, reducing the net flux of the material reaching the substrate. Additionally, rapid surface heating causes instantaneous vaporization, which further limits effective film growth and results in a decrease in thickness.^25^ Although increasing laser energy can enhance certain film properties, it may also introduce challenges related to film uniformity. At higher energy densities, re-evaporation effects become more significant, leading to the development of non-uniform and porous film structures. This porous morphology increases the active surface area and provides more sites for oxidation, which can enhance the gas sensing performance of the films.^26^

**Table 4:** Thickness of the Al_2_O_3_/AgO thin films at different energy densities of (a) *F* = 10.2 J cm^−2^, (b) *F* = 21.2 J cm^−2^, (c) *F* = 31 J cm^−2^, and (d) *F* = 40.8 J cm^−2^

Energy density (J cm^−2^)	Thickness (µm)
10.2	1.1037 ± 0.184
21.2	1.508 ± 0.59
31	4.210 ± 1.05
40.8	1.784 ± 0.22

In addition, the film thickness values presented in Fig. 6 were determined as the average of multiple measurements obtained from five different cross-sectional FE-SEM images. The corresponding error bars represent the standard deviation of these measurements, reflecting the variation in thickness across different areas of the films.

The presence of relatively large error bars in some samples indicates noticeable thickness non-uniformity, which is consistent with the porous and irregular morphology observed at higher laser energy densities. This behavior is inherent in PLD processes, in which localized redeposition and re-evaporation can lead to spatial variations in film growth. Therefore, the reported thickness values should be considered statistically representative averages rather than single-point measurements, providing a more reliable interpretation of the thickness evolution with laser energy density.

**Fig. 7:**
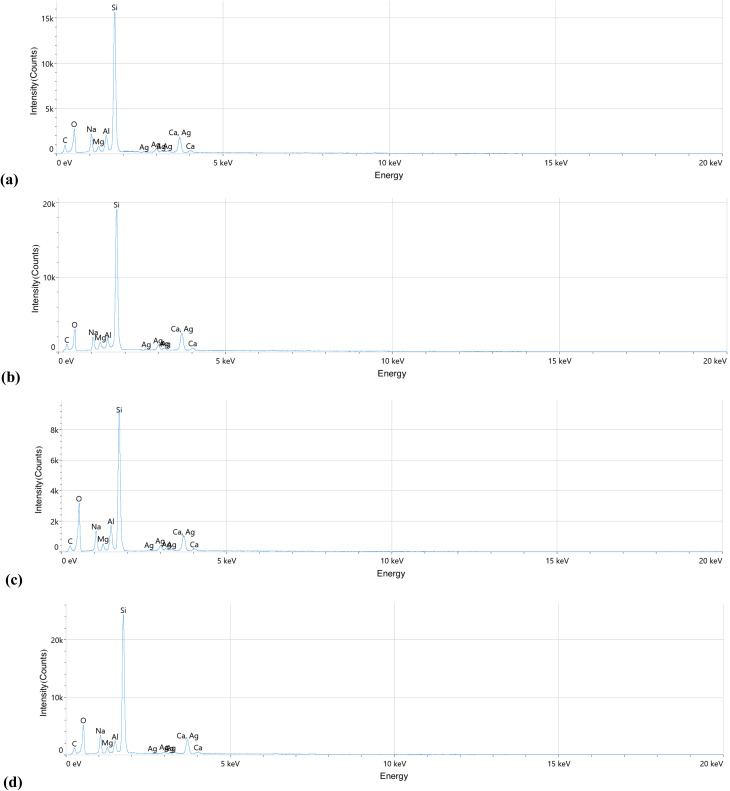
EDS patterns of the Al_2_O_3_/AgO samples at different energy densities of (a) *F* = 10.2 J cm^−2^ (b) *F* = 21.2 J cm^−2^, (c) *F* = 31 J cm^−2^, and (d) *F* = 40.8 J cm^−2^.

Fig. 7 illustrates the atomic emissions of Al, Ag, and O in Al_2_O_3_/AgO films deposited at different energy densities. Higher energy density leads to increased plasma density, which facilitates the incorporation of more Al and Ag atoms into the growing Al_2_O_3_/AgO films. This results in a higher atomic ratio of Al, Ag, and O, as well as an increase in the overall weight of the deposited material,^27,28^ as shown in Table 5. The increase in the oxidation of Al_2_O_3_/AgO films during PLD at higher laser intensities enhances energy input, promoting a more effective reaction with oxygen during the deposition process. The increase in oxidation in Al_2_O_3_/AgO films with higher laser intensity is likely due to enhanced energy input, promoting oxygen incorporation and facilitating oxidation during deposition.^28,29^ At this very high energy density (40.8 J cm^−2^) for Al_2_O_3_/AgO, a phenomenon known as the “ablation threshold” reduces the amount of material reaching the substrate, resulting in a decrease in both the deposited mass and the atomic percentage. It should be noted that the EDX spectrum presented in Fig. 7 is a background-subtracted spectrum, where the continuous background signal is removed during the analysis process to enhance the clarity and resolution of the characteristic elemental peaks. This processing step is commonly applied in EDX analysis to improve the accuracy of elemental identification and to minimize noise contributions from the substrate and measurement system. Furthermore, the detected peaks (Si, O, C, Na, Mg, and Ca) correspond to the typical components of the glass substrate due to the finite interaction volume of the electron beam, which can penetrate through thin films and partially excite the underlying glass substrate. This effect becomes more pronounced for relatively thin or porous films,^30^ as observed in the present study.

**Table 5:** Weight and atomic percentage of the elements in the prepared Al_2_O_3_/AgO

Energy density (J cm^−2^)	Elements	Atomic%	Weight%
*F* = 10.2	C	14.5	10.4
	O	44.5	25.1
	Na	7.3	8.0
	Mg	2.3	2.7
	Al	2.1	2.4
	Si	25.3	42.8
	Ca	2.3	6.6
	Ag	0.7	2.0
*F* = 21.2	C	12.6	9.3
	O	47.8	27.2
	Na	7.3	8.0
	Mg	2.2	2.6
	Al	2.5	3.1
	Si	22.8	38.8
	Ca	2.5	7.1
	Ag	1.3	3.9
*F* = 31	C	13.9	10.1
	O	51.9	29.6
	Na	6.3	7.3
	Mg	1.5	1.8
	Al	3.8	5.2
	Si	18.8	35.5
	Ca	2.2	6.4
	Ag	1.6	4.1
*F* = 40.8	C	11.5	8.0
	O	53.0	30.2
	Na	6.7	7.9
	Mg	2.5	2.8
	Al	2.4	3.0
	Si	20.2	36.7
	Ca	3.3	9.7
	Ag	0.4	1.7

**Fig. 8:**
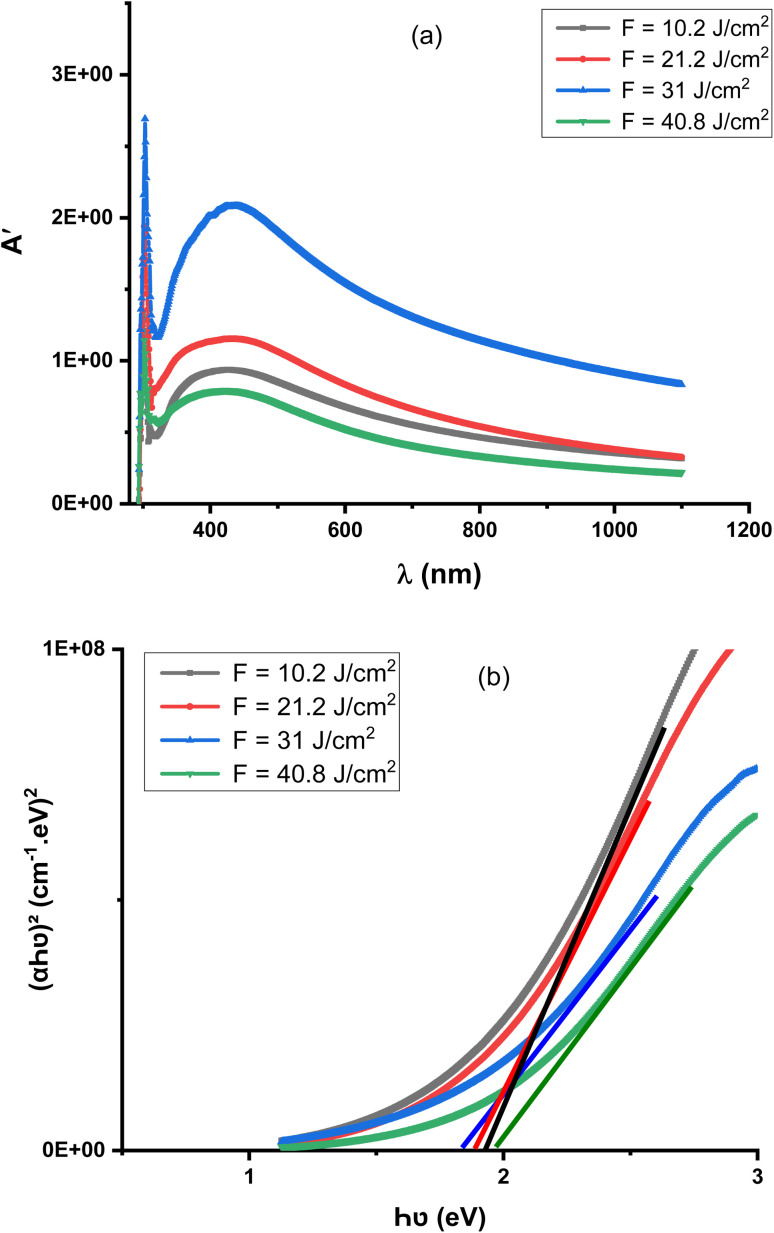
(a) Absorbance as a function of wavelength and (b) (*αhν*)^2^ as a function of (*hν*) for the Al_2_O_3_/AgO film at different energy densities of *F* = 10.2 J cm^−2^, *F* = 21.2 J cm^−2^, *F* = 31 J cm^−2^, and *F* = 40.8 J cm^−2^.

Fig. 8(a) illustrates the optical absorption spectra of the Al_2_O_3_/AgO thin films, which exhibit strong absorbance in the ultraviolet (UV) region, followed by a sharp decrease in the visible range. This behavior suggests limited long-range crystallinity and indicates that the films possess a predominantly disordered or nanostructured nature. The observed optical response is strongly dependent on the laser energy density employed during deposition.

At a laser energy density of 21.2 J cm^−2^, a noticeable increase in absorbance is observed, which can be attributed to enhanced ablation efficiency, leading to increased film thickness and improved surface coverage. In contrast, at a higher energy density (40.8 J cm^−2^), the absorbance decreases significantly. This reduction is consistent with the thinner films observed in Fig. 5 and can be associated with re-evaporation effects and reduced material accumulation at elevated laser energies.

Fig. 8(b) and Table 6 present the variation in the optical band gap (*E*_g_), which ranges from 1.821 to 1.967 eV. These values are in good agreement with previously reported results for similar composite systems.^31,32^ A gradual decrease in *E*_g_ is observed as the laser energy density increases from 10.2 to 31 J cm^−2^. This trend can be explained by the increase in crystallite size and the corresponding reduction in quantum confinement effects, which leads to a narrowing of the band gap.^22^ However, at the highest energy density (40.8 J cm^−2^), the optical band gap increases again. This behavior is attributed to a reduction in effective particle size and possible changes in film morphology, such as increased porosity or defect density, which can influence the electronic structure. Additionally, enhanced disorder and localized states at higher energies may contribute to the observed widening of the band gap.

**Table 6:** Optical energy gap (*E*_g_) values of the Al_2_O_3_/AgO film at different energy densities of (a) *F* = 10.2 J cm^−2^, (b) *F* = 21.2 J cm^−2^, (c) *F* = 31 J cm^−2^, and (d) *F* = 40.8 J cm^−2^

Energy density (J cm^−2^)	*E*_g_ (eV)
*F* = 10.2	1.92
*F* = 21.2	1.87
*F* = 31	1.82
*F* = 40.8	1.96

The Hall coefficient (*R*_H_), carrier concentration (*n*_H_), carrier mobility (*µ*_H_), and conductivity type of Al_2_O_3_/AgO thin films were systematically evaluated at different pulsed laser energy densities (10.2, 21.2, 31, and 40.8 J cm^−2^) using the Hall effect technique. Measurements were carried out using an HMS-3000 system (Ecopia, Korea) in the Van der Pauw configuration at room temperature under a magnetic field of 0.55 T. To ensure reliable ohmic contact, the indium electrodes were deposited in the four corners of the samples. This configuration enables the accurate determination of the Hall coefficient, carrier concentration, mobility, and resistivity.

As summarized in Table 7, all samples exhibited a negative Hall coefficient, indicating n-type conductivity. This behavior can be attributed to the presence of oxygen vacancies acting as donor-like defects, which are promoted under specific deposition conditions and laser energy densities. These vacancies introduce free electrons into the conduction band, thereby enhancing n-type conduction.

**Table 7:** Hall effect measurements for films at different energy densities of (a) *F* = 10.2 J cm^−2^, (b) *F* = 21.2 J cm^−2^, (c) *F* = 31 J cm^−2^, and (d) *F* = 40.8 J cm^−2^

Energy density (J cm^−2^)	*R*_H_ (cm^3^ C^−1^)	*n*_H_ (cm^−3^)	*µ*_H_ (cm^2^ V^−1^ s^−1^)	*σ*_RT_ (Ω cm^−1^)^−1^	Type (P/N)
10.2	−6.55 × 10^2^	9.53 × 10^15^	1.72 × 10^3^	2.63	N
21.2	−5.41 × 10^2^	1.15 × 10^16^	1.50 × 10^3^	2.77	N
31	−1.05 × 10^2^	5.93 × 10^16^	1.71 × 10^3^	1.62 × 10^1^	N
40.8	−4.95 × 10^2^	1.26 × 10^16^	1.36 × 10^3^	2.74	N

The Hall coefficient showed relatively minor variation with increasing laser energy density, suggesting that the dominant conduction mechanism remained unchanged across all samples. However, the carrier concentration (*n*_H_) exhibited a clear dependence on laser energy, increasing progressively and reaching a maximum of 31 J cm^−2^. This enhancement is attributed to improved crystallinity and reduced grain boundary density, which facilitate charge carrier generation and transport.

At a higher energy density (40.8 J cm^−2^), a slight reduction in carrier concentration was observed. This behavior is most likely associated with excessive laser-induced re-evaporation of material species, particularly silver and oxygen, leading to increased defect density and the formation of additional grain boundaries that act as carrier trapping and scattering centers.^33,34^ The carrier mobility (*µ*_H_) reached its maximum value at 21.2 J cm^−2^, indicating an optimal balance between crystallinity and defect density. At higher laser fluences, mobility decreased due to enhanced carrier scattering caused by structural imperfections, such as grain boundaries and oxygen vacancies.^34,35^ Consequently, the electrical conductivity followed a similar trend, increasing with laser energy up to 21.2 J cm^−2^ as a result of the combined improvement in carrier concentration and mobility, and then decreasing at 40.8 J cm^−2^ due to the detrimental effects of excessive energy input.

Overall, the results demonstrate that laser energy density plays a critical role in tuning the electrical properties of Al_2_O_3_/AgO thin films. Although moderate energy densities enhance crystallinity and charge transport, excessive energy leads to defect-dominated behavior, particularly through increased oxygen vacancy formation and grain boundary density, ultimately resulting in the degradation of electrical performance.

**Fig. 9:**
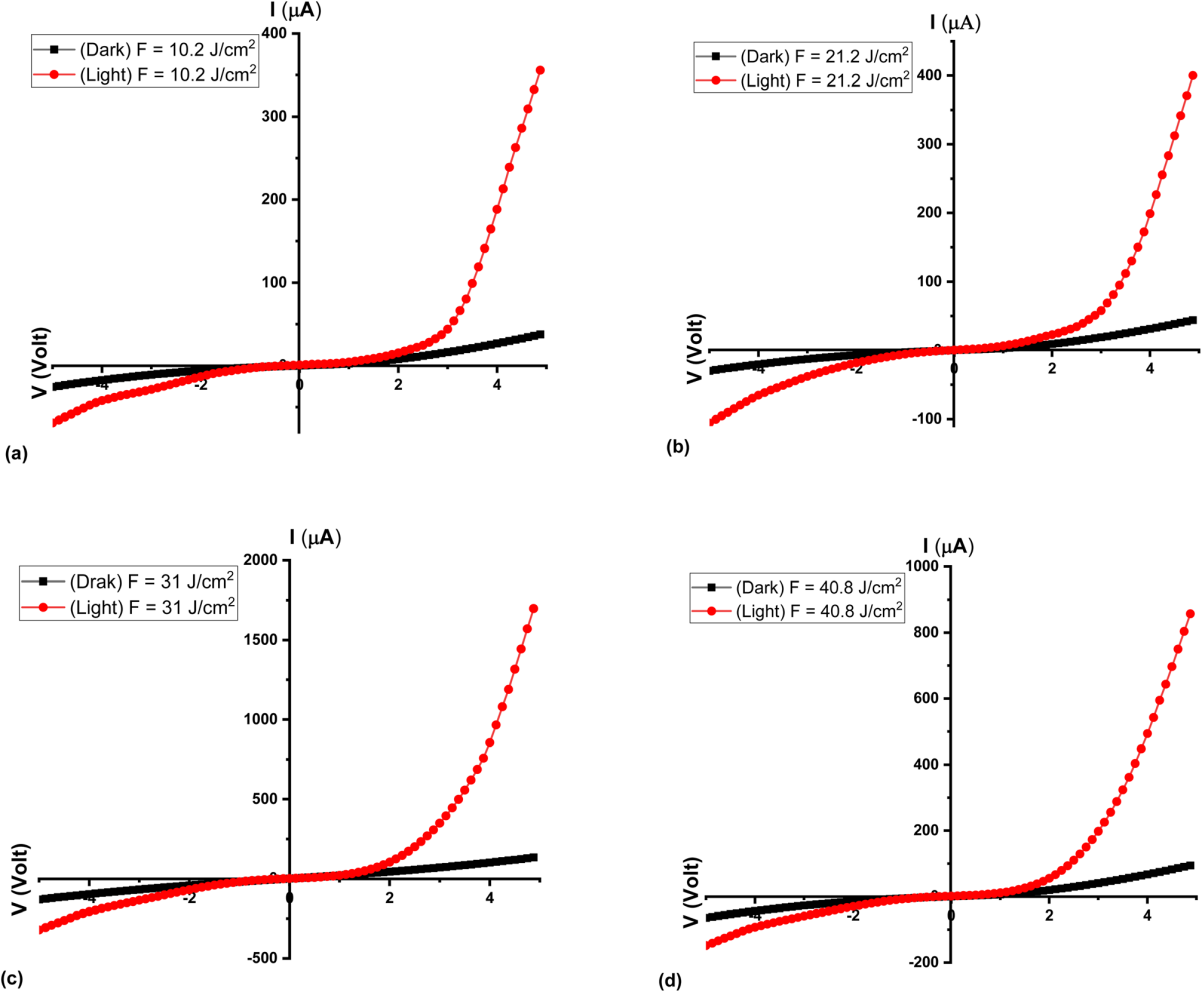
*I*–*V* characteristics for the Al_2_O_3_/AgO thin films at different energy densities of (a) *F* = 10.2 J cm^−2^ (b) *F* = 21.2 J cm^−2^, (c) *F* = 31 J cm^−2^, and (d) *F* = 40.8 J cm^−2^.

Fig. 9 illustrates the current–voltage (*I*–*V*) characteristics of Al_2_O_3_/AgO thin films deposited at different laser energy densities (10.2, 21.2, 31, and 40.8 J cm^−2^), measured at 308 K under both dark (black curves) and illuminated (red curves) conditions. A significant enhancement in photoconductivity is observed under illumination, which can be attributed to the increased generation of electron–hole pairs and the consequent reduction of potential barriers at grain boundaries, thereby facilitating charge transport.

Among all samples, the film deposited at 21.2 J cm^−2^ exhibits the highest forward bias current, indicating superior electrical transport properties at this energy density. With increasing applied voltage, the current increases accordingly and approaches an exponential trend under forward-bias conditions. In this regime, the current is predominantly governed by diffusion processes, where charge carriers move from regions of high concentration to low concentration, in accordance with Fick's law. This diffusion current dominates the recombination current, which otherwise reduces the number of free carriers through electron–hole recombination.

Furthermore, the increase in applied voltage leads to a reduction in the internal potential barrier and a narrowing of the depletion region, thereby enhancing the injection of majority carriers and improving current flow. In contrast, the sample prepared at 10.2 J cm^−2^ exhibits a relatively lower forward current, which can be attributed to poorer crystallinity and a higher density of defects and grain boundaries that act as carrier trapping centers.

Under reverse bias conditions, the *I*–*V* characteristics reveal two distinct regions. Initially, the reverse current remains low due to the widening of the depletion region and the reduced availability of free carriers. As the reverse bias increases, a slight rise in current is observed, which can be associated with leakage currents and defect-assisted conduction mechanisms.

The observed improvement in current with increasing laser energy density up to 21.2 J cm^−2^ is closely related to enhanced crystallinity and increased grain size, which reduces grain boundary density and minimizes carrier scattering. This reduction in structural defects decreases the number of trapping centers, thereby facilitating more efficient charge transport.^36^ However, at a higher energy density (40.8 J cm^−2^), the current decreases despite increased energy input. This behavior is attributed to the onset of re-evaporation effects, which introduce additional structural defects and disorders, including increased oxygen vacancy imbalance and grain boundary density. These defects act as scattering and recombination centers, ultimately hindering carrier mobility and reducing electrical conductivity.

## 4 Gas sensing performance of Al_2_O_3_/AgO thin films

Al_2_O_3_/AgO thin films were deposited *via* PLD at room temperature using varying laser fluences (10.2, 21.2, 31, and 40.8 J cm^−2^) and tested as gas sensors for NO_2_ (150 ppm) and H_2_S (200 ppm) over a temperature range of 40–250 °C. Resistance changes were monitored to evaluate sensing behavior (Fig. 12 and 13).

### 4.1 Response and recovery times

Response and recovery times were systematically evaluated for all samples across different laser energy densities and operating temperatures, as summarized in Table 8. It is clearly observed that, in most cases, the recovery time is longer than the response time. This behavior can be attributed to the relatively slower desorption kinetics of gas molecules from the sensor surface compared to the adsorption process. The rapid response is associated with the fast interaction between the target gas molecules and the active surface sites, leading to immediate charge transfer and a corresponding change in electrical resistance. In contrast, the recovery process requires the complete removal of adsorbed species and the restoration of the initial surface state, which is inherently slower due to stronger chemisorption bonding and possible surface trapping effects.

**Table 8:** Sensitivity, response time, and recovery time for the Al_2_O_3_/AgO films deposited at different energy densities (10.2, 21.2, 31, and 40.8 J cm^−2^) and at various operating temperatures

Energy density (J cm^−2^)	*T* (°C)	S–NO_2_ (%)	Res. time (s)	Rec. time (s)	S–H_2_S (%)	Res. time (s)	Rec. time (s)
10.2	40	74.84	14.94	15.57	63.6	10.53	21.33
	150	9.09	8.82	14.85	25.683	16.11	22.32
	250	51.48	22.68	19.53	40	16.92	25.29
21.2	40	6.5	19.71	21.51	47.88	7.92	19.62
	150	30.5	16.02	16.38	5.06	15.93	11.7
	250	45.1	14.49	18	55.26	16.02	20.97
31	40	16.1	10.53	31.23	8.41	15.57	20.7
	150	0.9	21.6	20.61	9.46	14.4	14.13
	250	37.5	14.04	17.64	37.03	19.62	17.1
40.8	40	18.13	16.74	15.48	14.28	10.8	20.16
	150	23.4	20.88	14.85	20.74	19.35	22.59
	250	29.7	18	21.06	62.067	7.83	18.27

This asymmetry between response and recovery times is characteristic of chemisorption-dominated sensing mechanisms, where gas molecules form relatively strong bonds with surface-active sites, particularly at elevated operating temperatures. Furthermore, recovery dynamics are influenced by factors such as surface defect density, grain boundary distribution, and oxygen vacancy concentration, all of which affect the adsorption–desorption equilibrium and the activation energy required for gas release.

### 4.2 Effect of sensor operation temperature

**Fig. 10:**
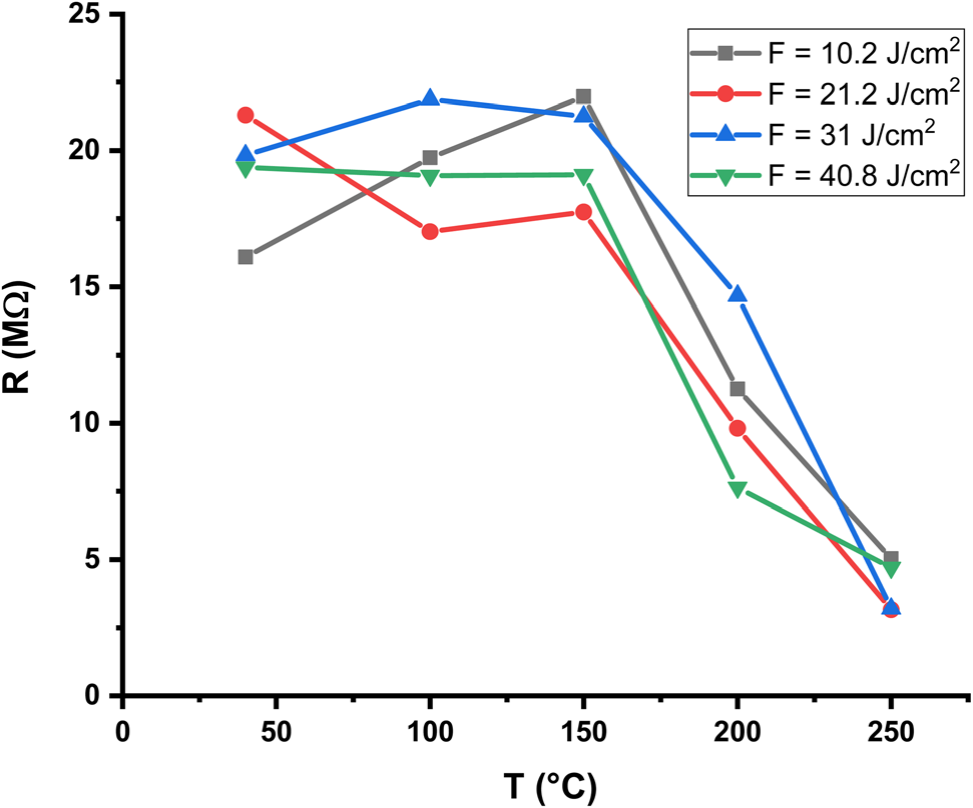
Resistance of the Al_2_O_3_/AgO thin films for NO_2_ gas as a function of temperature at different laser energy densities (10.2, 21.2, 31, and 40.8 J cm^−2^).

**Fig. 11:**
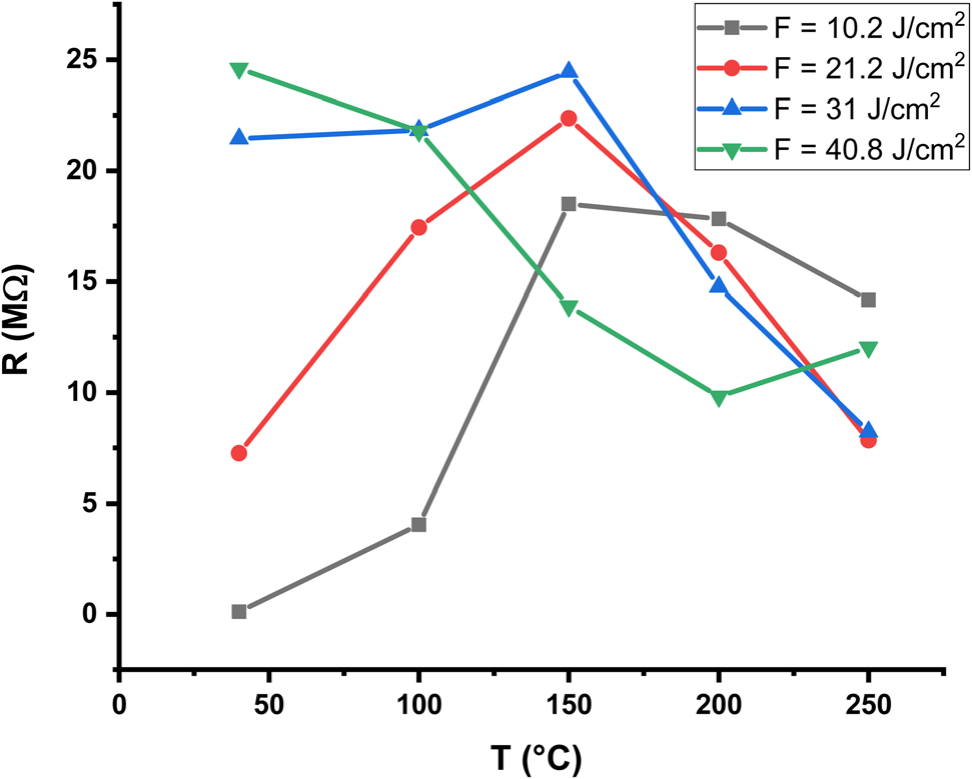
Resistance of the Al_2_O_3_/AgO thin films for H_2_S gas as a function of temperature at different laser energy densities (10.2, 21.2, 31, and 40.8 J cm^−2^).

The temperature dependence of the baseline resistance of the Al_2_O_3_/AgO thin films prepared at different laser fluences (10.2, 21.2, 31, and 40.8 J cm^−2^) is shown in Fig. 10 and 11. The measurements were carried out in air prior to gas exposure in order to evaluate the thermal behavior of the films and establish the baseline resistance of the sensors. In all cases, the resistance decreases with increasing temperature, which is characteristic of semiconducting materials. This behavior can be attributed to thermally activated charge transport, where increasing temperature enhances carrier excitation and facilitates conduction across grain boundaries, leading to lower resistance.

Although all films exhibit the same general semiconducting trend, noticeable differences are observed in the absolute resistance values and in the rate of resistance decrease. At lower temperatures, the films deposited at intermediate laser fluences (21.2 and 31 J cm^−2^) exhibit higher resistance than those prepared at 10.2 and 40.8 J cm^−2^. This behavior is likely associated with microstructural variations, including differences in grain-boundary density, film compactness, and defect concentration, all of which are strongly affected by laser energy density during PLD. At higher temperatures, the resistance values of all samples become closer, indicating that thermal activation becomes the dominant factor governing charge transport.

Small deviations observed between repeated measurements for the same sample may be attributed to surface-related effects, such as adsorbed oxygen or moisture, slight surface aging of silver oxide, and minor variations in electrode–film contact. Despite these variations, the results consistently confirm the semiconducting nature of the Al_2_O_3_/AgO films and demonstrate that laser fluence is an effective parameter for tuning the baseline electrical resistance of the sensor films.

**Fig. 12:**
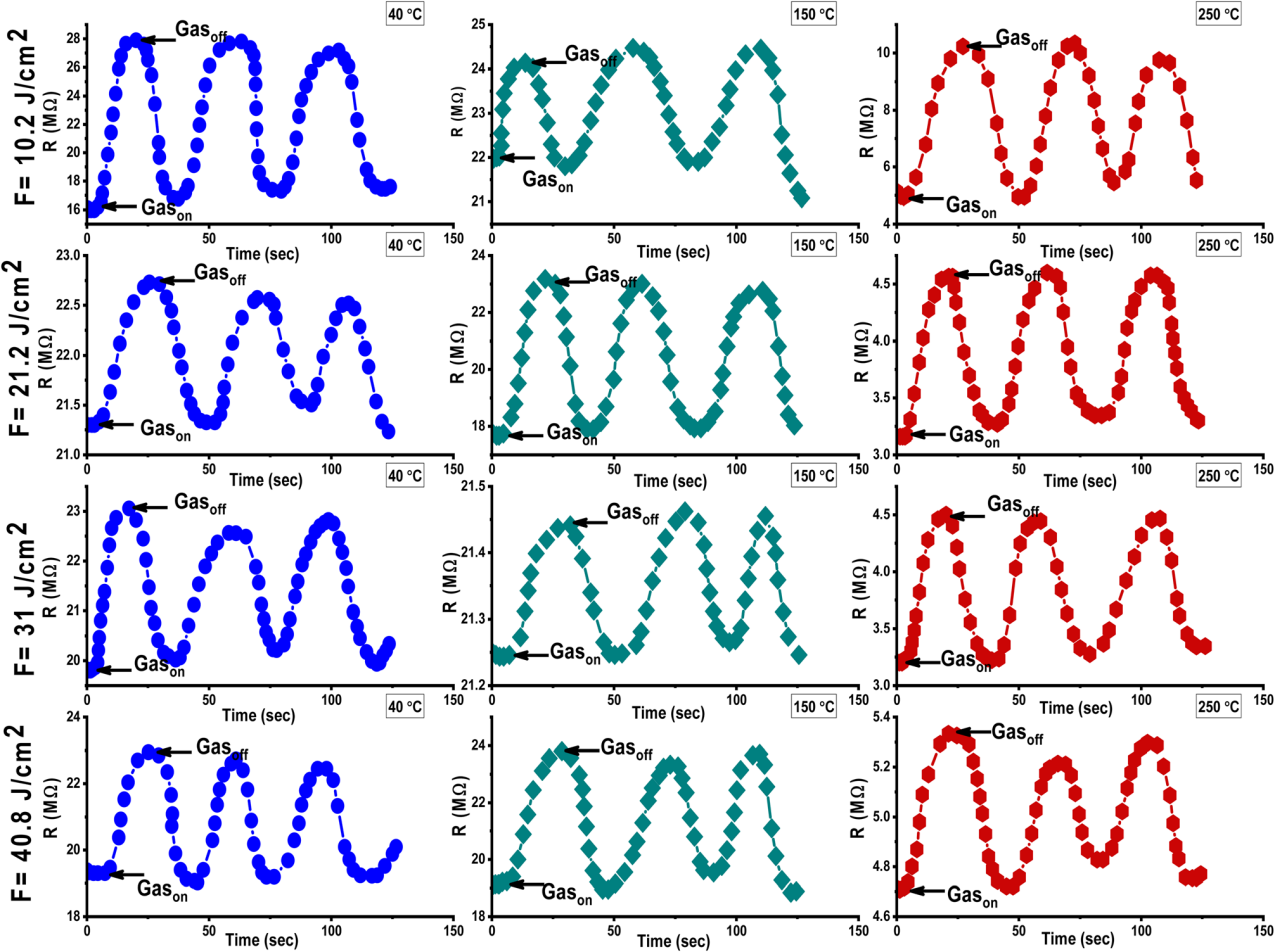
Resistance variation for the Al_2_O_3_/AgO thin films fabricated at different energy densities (10.2, 21.2, 31, and 40.8 J cm^−2^) and at various operating temperatures against NO_2_ gas.

**Fig. 13:**
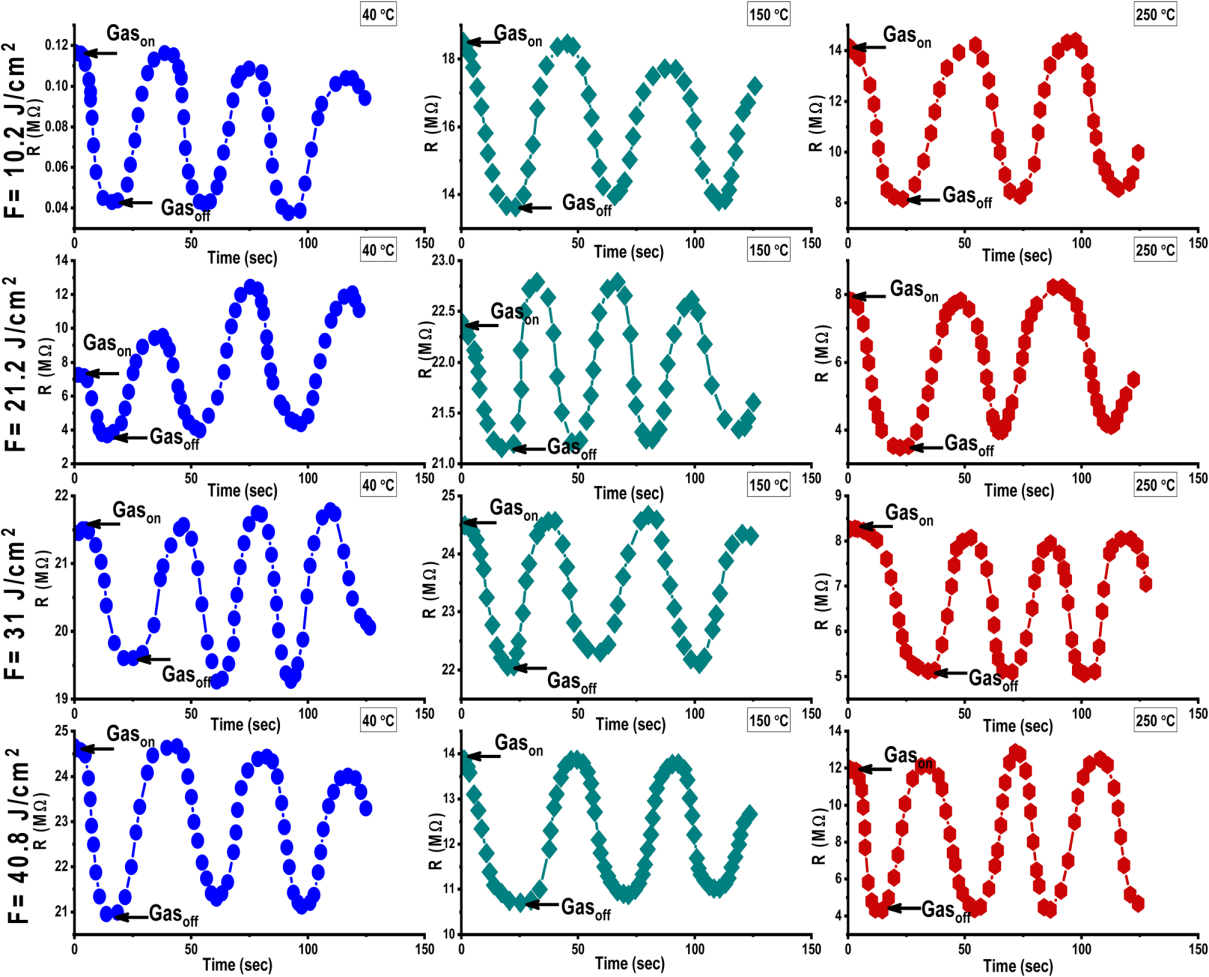
Resistance variation for the Al_2_O_3_/AgO thin films fabricated at different energy densities (10.2, 21.2, 31, and 40.8 J cm^−2^) and at various operating temperatures against H_2_S gas.

Fig. 12 and 13 show how the sensitivity of Al_2_O_3_/AgO films fabricated at different energy densities (10.2, 21.2, 31, and 40.8 J cm^−2^) varies with the operating temperature when exposed to NO_2_ and H_2_S gases. As shown in Table 8, sensitivity increases with temperature due to enhanced gas adsorption, which requires activation energy,^37^ reaching a maximum at 250 °C (the optimum operating temperature), where gas adsorption and reaction rates are highest.^38^

For H_2_S, a reducing gas, sensitivity is limited to below 150 °C due to weak interaction with surface oxygen ions (O^−^). At 250 °C, H_2_S reacts effectively, releasing electrons as follows:

1 H_2_S + 3O^−^(ads) → H_2_O(g) + SO_2_ + 3e^−^.

This reaction lowers resistance in the n-type Al_2_O_3_/AgO sensor. Hence, temperature control is crucial for optimal sensing, and the high melting point of Al_2_O_3_ (>2000 °C) ensures structural stability during high-temperature operation.^39^

### 4.3 Effect of increasing energy density on film sensitivity

The definition of sensitivity depends on the type of gas (oxidizing or reducing). If the gas is reducing, the sensitivity is given by the following equation:2
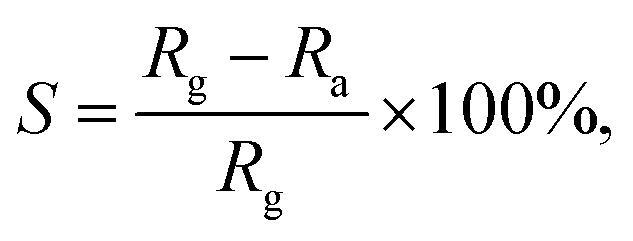
where *R*_a_ and *R*_g_ represent resistance in the presence of air and gas, respectively. However, in the case of the presence of an oxidizing gas, the sensitivity is given by the following equation:3
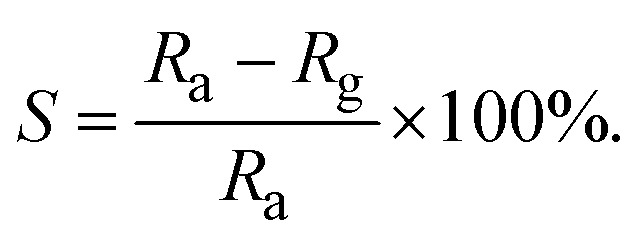


#### 4.3.1 Exposure to NO_2_ gas

The sensitivity, response, and recovery times of Al_2_O_3_/AgO sensors exposed to 150 ppm of NO_2_ gas were evaluated for films deposited at different energy densities (10.2, 21.2, 31, and 40.8 J cm^−2^). As shown in Fig. 12, exposure to NO_2_ led to an increase in resistance due to electron withdrawal by NO_2_ molecules, which is consistent with the behavior of n-type semiconductors. This occurs as NO_2_ molecules interact with surface-adsorbed O^−^ ions, drawing electrons from the conduction band and widening the depletion layer, which reduces conductivity.^3,5^

The experimental data showed that sensitivity decreased as laser energy increased from 10.2 to 31 J cm^−2^ (Table 8). Structural analysis (XRD and FE-SEM) revealed that this was due to a larger grain size and higher crystallinity, leading to fewer grain boundaries, which are critical active sites for gas adsorption.

Although the surface roughness slightly increased, it was not sufficient to compensate for the loss of adsorption sites, resulting in a decrease in sensor performance at higher fluences.

At the highest deposition energy of 40.8 J cm^−2^, the kinetic energy of the ablated particles increases, leading to re-evaporation and non-uniform grain growth. These conditions promote the formation of crystalline defects, particularly oxygen vacancies (V_o_), which act as active sites for gas adsorption. Importantly, the high electron affinity of Ag/AgO further enhances the electron-withdrawing effect of NO_2_, amplifying depletion layer widening and contributing to the marked increase in resistance. Thus, the synergistic effect of oxygen vacancies and the strong electron affinity of silver improves sensitivity at the highest energy density, as illustrated in Table 8.

#### 4.3.2 Exposure to H_2_S gas

When exposed to air, oxygen molecules adsorb onto the Al_2_O_3_/AgO surface, capturing electrons from the conduction band and forming species like O_2_^−^, O^−^, and O_2_^2−^. This widens the depletion layer.^40^ Upon exposure to 200 ppm of H_2_S, these oxygen species react with H_2_S, releasing trapped electrons and reducing the resistance of the sensor. H_2_S also undergoes surface decomposition into HS and sulfur (S).^6^

As shown in Fig. 13, the sensor exhibited a rapid and repeatable response to H_2_S at 40 °C, 150 °C, and 250 °C, with the resistance returning to baseline after the gas was removed. However, sensitivity decreased at higher energy densities (10.2–31 J cm^−2^) due to grain growth and the resulting reduction in grain boundaries, which limited the number of adsorption sites.

At 40.8 J cm^−2^, the formation of oxygen vacancies and grain boundaries provided new active sites, enhancing H_2_S adsorption and electron transfer. As a reducing gas, H_2_S increases free electrons in the n-type film, improving conductivity. This explains the enhanced sensitivity at the highest energy density.

In this case, the high electron affinity of silver plays a dual role: it facilitates electron withdrawal under oxidizing conditions, and it magnifies the release of electrons during the reduction process induced by H_2_S. This stronger modulation of the conduction band improves conductivity changes and explains the enhanced sensitivity and reproducibility of the sensor at the highest energy density.

### 4.4 Sensing mechanism

All tested samples exhibited n-type semiconductor behavior, characterized by an increase in resistance upon exposure to NO_2_ and a decrease upon exposure to H_2_S. This behavior aligns with the general sensing mechanism of metal oxide semiconductors, which is dominated by changes in surface resistance. Atmospheric oxygen molecules are adsorbed onto the film surface and ionized by trapping electrons from the conduction band, forming species such as O_2_^−^ and O^−^. This leads to the development of a surface depletion layer and the creation of potential barriers that hinder charge carrier transport between nanoparticles.

When exposed to reducing gases like H_2_S, these chemisorbed oxygen species react with the gas molecules, resulting in the release of trapped electrons back into the conduction band and consequently decreasing the resistance. In contrast, oxidizing gases, such as NO_2_, extract additional electrons, thereby deepening the depletion layer and increasing resistance. These dynamic changes in surface charge density modulate the electrical conductivity of the sensors.^41^

## 5 Comparative analysis of gas sensing performance with recent literature

To further assess the sensing capability of the present Al_2_O_3_/AgO thin films, their performance was compared with representative metal-oxide-based gas sensors reported in the literature, as summarized in Table 9. For H_2_S sensing, the present Al_2_O_3_/AgO film achieved 74.20% sensitivity at 200 ppm and 250 °C, with response/recovery times of 24.84/16.02 s. This performance is clearly superior to that of the Cr_2_O_3_–γFe_2_O_3_ (1 : 2) nanocomposite, which exhibited a response of 16.1 toward 50 ppm H_2_S at 200 °C with 16.2/48.6 s response/recovery times. Notably, that study attributed the sensing behavior to an n-type mechanism governed by adsorbed oxygen species and enhanced performance with increasing Fe_2_O_3_ content, yet the recovery remained substantially slower than that of the present film. Likewise, amorphous IGZO nanofibers showed a response of 40.5 toward 100 ppm H_2_S at 250 °C, where the authors linked the improvement to the large surface area of the nanofiber morphology, the absence of grain-boundary limitations, and the contribution of oxygen-vacancy-related active sites. However, the present Al_2_O_3_/AgO film still provides a higher sensitivity under a higher target-gas concentration while maintaining short recovery kinetics. Within the remaining H_2_S comparators listed in Table 9, CuO/SnO_2_ offers a somewhat higher nominal sensitivity (85.71%) but suffers from very slow kinetics (100/109 s), whereas NiO exhibits both lower sensitivity (28.8) and a much slower response (108/47 s) at a significantly higher operating temperature (400 °C). Although Ag/WO_3_/rGO shows a fast initial response (8 s), it exhibits longer recovery (38 s). Accordingly, the sensor offers one of the most attractive time-domain trade-offs among the compared H_2_S systems.

**Table 9:** Comparison of the gas sensing performance of the Al_2_O_3_/AgO thin films with previously reported metal oxide-based gas sensors under comparable operating conditions

Material	Gas (concentration)	*T* (°C)	Sensitivity (%)	Response time (s)/recovery time (s)	References
Al_2_O_3_/AgO	H_2_S (200 ppm)	250	74.20	24.84/16.02	This work
Cr_2_O_3_–γFe_2_O_3_ (1 : 2)	H_2_S (50 ppm)	200	16.1	16.2/48.6	42
IGZO	H_2_S (100 ppm)	250	40.5	—	43
CuO/SnO_2_	H_2_S (50 ppm)	200	85.71	100/109	44
NiO	H_2_S (200 ppm)	400	28.8	108/47	44
Ag/WO_3_/rGO	H_2_S (100 ppm)	150	65.8	8/38	44
Al_2_O_3_/AgO	NO_2_ (150 ppm)	250	55.56	11.61/20.43	This work
TiO_2_ NTs/rGO	NO_2_ (100 ppm)	RT	14.9	18/33	45
NiO	NO_2_ (100 ppm)	200	45.6	13/146	46
WO_3_	NO_2_ (100 ppm)	200	97	12/412	46

For NO_2_ sensing, the Al_2_O_3_/AgO film delivered 55.56% sensitivity at 150 ppm and 250 °C with 11.61/20.43 s response/recovery times, demonstrating rapid and reversible operation. In comparison, the TiO_2_ NTs/rGO heterojunction sensor operated at room temperature and showed a highly sensitive response of 19.1 to 1 ppm NO_2_, with improved 18/33 s response/recovery times after rGO incorporation. The reported enhancement was attributed to the increased specific surface area, a higher amount of chemisorbed oxygen, and the formation of TiO_2_/rGO heterojunctions that facilitated charge separation and gas adsorption. Although room-temperature operation is an advantage of this system, the present Al_2_O_3_/AgO film maintains competitive kinetics at a much higher NO_2_ concentration and under thermally activated sensing conditions. A similar observation applies to the porous NiO sensor, which showed 45.6% response to 100 ppm NO_2_ at 200 °C with 13/146 s response/recovery times. In that study, the porous nanosheet morphology and oxygen-deficient regions were identified as the key sources of reactivity, yet the sensor still exhibited a very slow recovery process compared with the present film. Thus, even though some literature sensors may show a higher response under specific conditions, the Al_2_O_3_/AgO thin films reported herein distinguish themselves by combining relatively high sensitivity and fast recovery, which are crucial for practical sensing applications involving repeated on/off operation, reduced drift, and improved operational stability.

Overall, the comparison confirms that the present Al_2_O_3_/AgO thin films provide a well-balanced sensing profile rather than merely a high isolated response value. Their enhanced performance is consistent with the structural and morphological characteristics observed in this study, particularly the synergistic role of AgO redox activity, the thermally stable Al_2_O_3_ matrix, and the defect-rich/porous microstructure produced under optimized PLD conditions. These features collectively promote efficient adsorption–reaction–desorption kinetics and explain why the present films outperform several previously reported metal oxide systems in terms of practical dynamic gas-sensing behavior.

## 6 Conclusion

This study shows that the laser energy density in pulsed laser deposition (PLD) plays a decisive role in shaping the structure, optical behavior, electrical transport, and gas-sensing response of Al_2_O_3_/AgO thin films. When the energy density was increased from 10.2 to 31 J cm^−2^, the films became more crystalline with larger grains, but the sensitivity decreased because of the loss of grain boundary adsorption sites. At the highest fluence of 40.8 J cm^−2^, however, the creation of oxygen vacancies and nanoclusters increased porosity and surface roughness, which introduced new active sites and improved the response.

All films behaved as n-type semiconductors, with resistance increasing under NO_2_ and decreasing under H_2_S exposure. The most favorable sensing temperature was found to be 250 °C, where adsorption and desorption processes reached their optimum balance, giving the highest response. The Al_2_O_3_ matrix provided the necessary thermal stability, protecting the films from degradation at elevated temperatures.

By directly linking deposition energy to microstructural evolution, defect formation, and sensing performance, this study addresses a gap not previously clearly examined in previous studies. The results suggest that the careful control of PLD energy density is an effective route for tailoring thin-film sensors and offers practical guidance for developing high-performance, thermally stable devices. Future studies may focus on defect engineering and doping strategies to further refine sensitivity and selectivity.

Future studies will focus on rigorous long-term stability, drift, and repeatability testing under varying humidity conditions to assess the commercial viability of the system.

Future studies will involve extending the measurements to sub-ppm and permissible exposure levels, where defect engineering and doping strategies may further enhance sensitivity and enable reliable detection at such trace concentrations.

We agree that future studies should focus on lower concentrations, which are closer to environmental and occupational exposure limits, and this point has been acknowledged in the revised manuscript.

Our findings show that laser energy density in PLD is a powerful tool for controlling the structure and gas-sensing behavior of Al_2_O_3_/AgO thin films, which aligns with and extends recent studies. Alzahrani *et al.* (2025) emphasized the role of silver in enhancing sensitivity,^47^ while Shen *et al.* (2025) and Kim *et al.* (2023) demonstrated improvements in NO_2_ sensing through Ag modification and porosity control.^48,49^ Similarly, Akbari-Saatlu *et al.* (2024) highlighted the impact of porosity on H_2_S detection at sub-ppb levels.^50^ In comparison, our study achieves comparable or superior enhancements without additional surface modification since oxygen vacancies, nanoclusters, and porosity are intrinsically tuned by laser fluence. This establishes a direct link between deposition energy, defect formation, and sensing response, providing a simpler and more systematic approach than previously reported.

## Author contributions

All authors contributed to the conception and design of this study.

## Conflicts of interest

The authors have no conflicts of interest.

## Data Availability

The paper contains the information that supports this study.
